# Higher sensitivity of female cells to ethanol: methylation of DNA lowers Cyp2e1, generating more ROS

**DOI:** 10.1186/s12964-020-00616-8

**Published:** 2020-07-11

**Authors:** Carlos G. Penaloza, Mayra Cruz, Gabrielle Germain, Sidra Jabeen, Mohammad Javdan, Richard A. Lockshin, Zahra Zakeri

**Affiliations:** 1 Queens College and Graduate Center, City University of New York, 65-30 Kissena Blvd, NSB E143, Flushing, NY 11367 USA; 2 Present Address: Chancellor’s Office, Leeward Community College, Pearl City, HI USA; 3 Queensborough Community College, City College of New York, Bayside, NY USA

**Keywords:** Sex differences, Cyp450, ROS, Cell death, Methylation

## Abstract

**Background:**

Cells taken from mouse embryos before sex differentiation respond to insults according to their chromosomal sex, a difference traceable to differential methylation. We evaluated the mechanism for this difference in the controlled situation of their response to ethanol.

**Methods:**

We evaluated the expression of mRNA for alcohol dehydrogenase (ADH), aldehyde dehyrogenases (ALDH), and a cytochrome P450 isoenzyme (Cyp2e1) in male and female mice, comparing the expressions to toxicity under several experimental conditions evaluating redox and other states.

**Results:**

Females are more sensitive to ethanol. Disulfiram, which inhibits alcohol dehydrogenase (ADH), increases cell death in males, eliminating the sex dimorphism. The expressions ADH Class 1 to 4 and ALDH Class 1 and 2 do not differ by sex. However, females express approximately 8X more message for Cyp2e1, an enzyme in the non-canonical pathway. Female cells produce approximately 15% more ROS (reactive oxygen species) than male cells, but male cells contain approximately double the concentration of GSH, a ROS scavenger. Scavenging ROS with N-acetyl cysteine reduces cell death and eliminates sex dimorphism. Finally, since many of the differences in gene expression derive from methylation of DNA, we exposed cells to the methyltransferase inhibitor 5-aza- 2-deoxycytidine; blocking methylation eliminates both the difference in expression of Cyp2e1 and cell death.

**Conclusion:**

We conclude that the sex-differential cell death caused by ethanol derives from sex dimorphic methylation of Cyp2e1 gene, resulting in generation of more ROS.

## Plain English summary

Cells isolated from embryonic female mice, even before the embryos have sexually differentiated, are more sensitive to ethyl alcohol (ethanol) than are cells from male mice. We have traced this difference to differing amounts of methylation, particularly in genes responsible for detoxification of such chemicals. Here we demonstrate that female mice use a subsidiary pathway to detoxify ethanol, producing more reactive oxidizing substances, while males have more reducing substances to counteract the oxidation. Interfering with either the production of reactive oxygen or with the differential methylation of the detoxifying genes eliminates the sex dimorphism. We conclude that alcohol produces toxic reactive oxidizing molecules, more so in females, because of differences in the regulation of detoxifying enzymes.

## Background

Cells derived from different sex embryos but similar organs respond to toxic stimuli in a sex dependent manner [1], and expression and regulation of select genes including members of the detoxifying cytochrome p450 (CYP) enzyme family, [1, 2] depends on the sex of cultured cells. These expression differences are partially regulated by DNA methylation [2].

Cytochrome P450 proteins, named for the absorption at 450 nm when binding carbon monoxide represent a very large superfamily, found in the genomes of virtually all organisms. The enzymes use electrons from NAD(P) H to catalyze activation of molecular oxygen, leading to region-specific and stereospecific oxidative attack of many substrates [3]. In eukaryotes, they are usually bound to the endoplasmic reticulum or inner mitochondrial membranes. The electron carrier proteins used for conveying reducing equivalents from NAD(P) H differ with subcellular localization [3]. CYPs are expressed in a sex specific manner in pigs and minipigs [4], and there are sex differences in the circadian variation of hepatic cytochrome P450 genes and corresponding nuclear receptors in mouse liver [5]. Sex-specific changes in certain cytochrome P450 proteins exist in response to insulin-dependent diabetes reflect sex-specific differences in growth hormone and triglyceride levels in the diabetic animals [6]. The inheritance mode of Quantitative Trait Loci (QTL), for which Cyp19a1 is a candidate gene, on chromosome 9 is sex specific [7]. Women show greater hepatic CYP3A activity than men [8]. Cyp1a1, Cyp2e1, and Cyp7b1 are dimorphically expressed and regulated in isolated cell cultures [1]. CYP1A2, CYP2A19, CYP3A22, CYP4V2, CYP2C36, CYP2E_1, and CYP2E_2 show significant sex differences in protein abundance [9]. Drug-Drug Interactions involving CYP3A4 substrates could potentially show sex dimorphism [10]. Tamargo et al. [11] have reported gender differences in the pharmacokinetics and pharmacodynamics of cardiovascular drugs for drug metabolizing enzymes and transporters. They described, among many metabolic differences, that CYP2B6 and CYP3A4 are more active in females, while CYP1A2 and CYP2E1 were more active in males. This dimorphism possibly affects manifestation of disease. We chose to explore the possibility by exploring Cytochrome P450 2E1 (Cyp2e1), which plays a role in ethanol-induced oxidant stress and is a minor but resolvable pathway of ethanol oxidation.

**Fig. 1:**
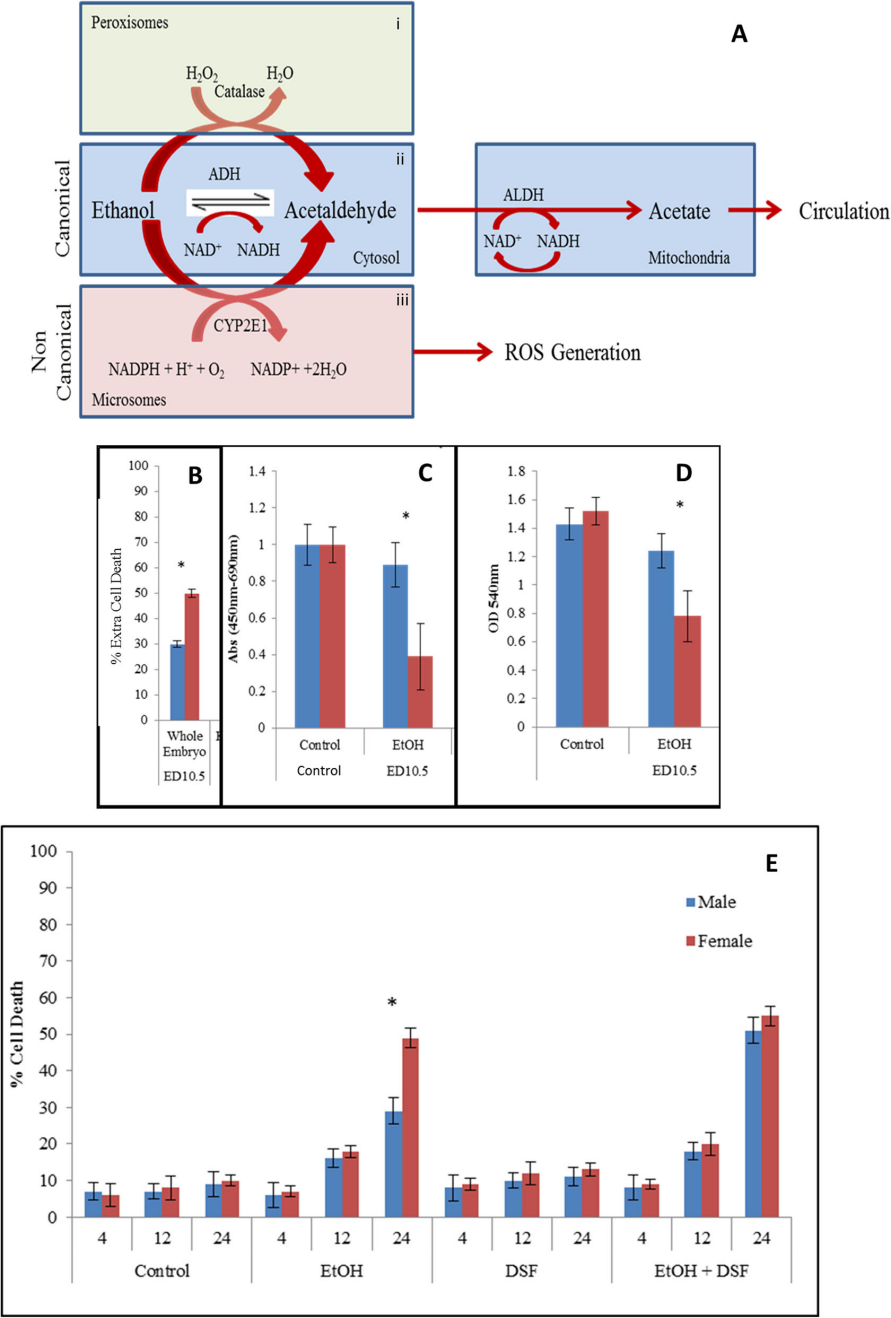
Cell viability after ethanol exposure is sex dimorphic in ED10.5 and this dimorphism is eliminated by inhibition of ALDH. (**a**) This cellular representation of alcohol metabolism shows the canonical alcohol metabolism pathway: ethanol enters the cell by diffusion and is metabolized by alcohol dehydrogenase in the cytoplasm to acetaldehyde. Aldehyde dehydrogenase (ALDH) further metabolizes the acetaldehyde to acetate, which is later used by the cell. The non-canonical alcohol metabolism pathway is similar to the canonical pathway, but the initial metabolic step is performed by Cyp2e1 on microsomes and results in generation of reactive oxygen species (ROS). Alcohol dehydrogenase (ADH) is the major enzyme pathway for oxidizing ethanol to acetaldehyde. (**b**) Cell death measured by trypan blue exclusion. In this and other figures, blue bars represent male cells and red bars, female cells. Cells from ED10.5 whole embryo in culture were exposed to 400 μM ethanol for 24 h. Data represent percentage total cell death for all conditions. These experiments were done in triplicate. Higher female cell sensitivity to ethanol is seen in cells. Asterisks (*) indicate *p* < 0.05 between male and female. The error bars represent S.D. (**c**) Measure of mitochondrial membrane integrity by WST-1 after exposure to 400 μM EtOH. Cells were seeded and incubated in 96-well dishes for the final hour of a 24-h exposure to EtOH with the Wst-1 compound and read at 450 nm with background subtracted at 690 nm. Male cells have higher WST-1 activity in the presence of EtOH for ED10.5 whole embryo cells. These experiments were done in triplicate, with asterisks (*) representing *p* < 0.05. (**d**) Confirmation of the WST-1 assay with MTT assay. Cell mitochondrial integrity after exposure to 400 μM EtOH for 24 h. Cells were supplemented with MTT stock for 2 h, and then the dye was solubilized and supernatants were read at 540 nm. Female cells are more sensitive than male cells to EtOH. These experiments were done in triplicate, with asterisks (*) representing *p* < 0.05. (E) Cell death measured by trypan blue exclusion. Cultured cells exposed to 400 μM ethanol, DSF or a combination. Higher female cell sensitivity to ethanol is seen in cells from ED10.5 whole embryo, but not in cells co-treated with DSF. Data represent percentage total cell death for each condition. These experiments were done in triplicate. Asterisks (*) represent *P* < 0.05

Alcohol is metabolized by two pathways in humans: the ADH (canonical) (Fig. 1A.ii) pathway accounts for the bulk of the metabolism, and the MEOS (Microsomal Ethanol Oxidizing System) (non-canonical) (Fig. 1a.iii) pathway which, at toxic alcohol levels, contributes to the increased rate of ethanol metabolism [12–14]. There are also individual differences in ADH isoenzymes [12, 15, 16]. Women have a higher likelihood of developing liver cirrhosis than men [17]. Female Pregane X receptor-Humanized mice (hPXR) have higher basal alcohol dehydrogenase 1 and aldehyde dehydrogenase 2 allowing female hPXR mice to eliminate blood EtOH more rapidly than their male counterparts [18].

Cyp2e1 metabolizes several small, hydrophobic substrates and drugs [13, 14] such as ethanol. EtOH induces Cyp2e1 in human neuroblastoma SH-SY5Y cells [19]. Toxicity of these compounds is enhanced after induction of Cyp2e1, e.g., by ethanol treatment, and toxicity is reduced by inhibitors of Cyp2e1 or knockout of the gene in mice [13, 14]. Cyp2e1 is a minor pathway of ethanol oxidation as it catalyzes the two-electron oxidation of ethanol to acetaldehyde [20]; it theoretically can also oxidize acetaldehyde to acetate [13, 14]. Cyp2e1 generates O_2_·^−^and H_2_O_2_ during its catalytic cycle [21]. Because Cyp2e1 can generate ROS and the enzyme is elevated by chronic treatment with ethanol, Cyp2e1 may contribute to ethanol-induced oxidant stress and to ethanol-induced liver injury [13, 14]. Cyp2e1 inhibitors such as diallyl sulfide [22], blocked the lipid peroxidation and ameliorated the pathologic changes in ethanol-fed rats [13, 23].

Ethanol increases CYP2E1, largely by a posttranscriptional mechanism involving enzyme stabilization against degradation [14, 24]. Cyp2e1 induction by ethanol is regulated by p38 and ERK1/2 pathways in neurons. Patients with Parkinson’s Disease have more methylated CYP2E1 gene in their brain tissue compared to healthy brains [19]. We have previously shown that ethanol differentially induces the expression of Cyp2e1 between male and female cells; and that this difference in expression can be altered by DNA methylation [2]. Moreover, gene array analysis of sex dimorphic genes identified Dnm3tl as dimorphically expressed, further corroborating the potential role of DNA methylation in the regulation of sex dimorphic genes [1].

This evidence suggests that the linkage between Cyp2e1-derived oxidative stress, mitochondrial injury, and GSH homeostasis contributes to the toxic actions of ethanol, and that this mechanism is sex dimorphic. We explored this possibility by exploring the extent to which cells derived from mouse embryos taken before sex differentiation differed in response to EtOH, the amounts of Cyp enzymes present, the amounts of ROS generated by stress, and the extent to which blockage of methylation could eliminate the sex differences.

## Methods

### Mice and cells

CFW Swiss Webster from the Charles River Carworth Colony were purchased and maintained in our animal facility at Queens College. Male and female age matched (4–9 month) Swiss Webster mice were placed together overnight; the females were checked for vaginal plugs and separated setting the gestational time at day 0.5. They were maintained separately until the desired gestational time was reached.

At embryonic day 10.5, pregnant females were sacrificed by CO_2_ and cervical dislocation. Embryos were removed from the mother and placed in chilled sterile 1X PBS (Phosphate Buffered Saline). Each embryo was removed from its embryonic sac and placed in chilled Dulbecco’s Modified Eagle Medium (DMEM). The tissues were triturated by pipetting the tissue until it was completely dissociated. Suspended cells were collected by centrifugation at 3000 rpm and resuspended in media containing DMEM supplemented with 10% Fetal Bovine Serum (FBS), 100 U/mL penicillin, and 100 mg/mL streptomycin, and divided into several tissue culture plates and incubated at 37^o^ C in a humidified atmosphere with 5% CO_2_. Cells were grown for approximately 4 days or to 70% confluence before re-plating them. Medium was changed every 3 days. The cells were washed in PBS before feeding with fresh culture medium. At 70% confluency the culture dishes were trypsinized and cells were counted and plated at 500,000 cells/ 10 mm^2^. For sexing day 10.5 embryos, which do not yet have visible gonads, we used PCR. A small piece of tail was placed in a sterile PCR tube. DNA was isolated by digestion with 2 μl proteinase K (10 mg/ml) and 50 μl PCR-D buffer and incubating overnight at 65^o^ C. The enzyme was denatured at 95 °C for 10 min and 1 μl was transferred into a PCR tube, to which was added 21 μl dH_2_O, 25μlPCR Master Mix (Sigma), 2 μl MgCl_2_ (20 mM), 1 μl Primer Mix (25 pmol/μl of Zfy and Zfxya, 12.5 pmol/μl of Zfx primers). Primers for Zfy were 5′-CTCCTGATGGACAAACTTTACGTCTC and 3′-GCTGAGCCTCTTTGGTATCTGAGAAA; primers for Zfxya were 5′-GAGAGCATGGAGGGCCATG and 3′-GAGTACAGGTGTGCAGCTC;Zfx primers were 5′-CTCTGAAGAAGAGACAAGTT and 3′-CTGTGTAGGATCTTCAATC. PCR was run for 40 total cycles (1 Cycle = 94 °C for 45′, 60 °C for 25′, 72 °C for 1″) in the Eppendorf 2200 thermal cycler. The results were visualized on a 12% native polyacrylamide gel in Tris Buffer with EDTA TBE buffer using positive controls (pre-amplified male and female samples) and viewed by UV illumination. Male samples showed 2 bands, a 124 bp band for Zfy gene and a 134 bp band for gene Zfx and females showed a single band at 134 bp for Zfx gene [24]. Each embryo was used as an *N* = 1 in blind experiments, without knowing the sex of the embryo. Cells of each embryo were propagated and seeded at 5 × 10^5^/mL and exposed to ethanol. When the sexes of the blind cultures were determined, the results of each sample were pooled for statistical determination.

Dulbecco’s Modified Eagle medium (DMEM) (catalog no. 12800–017; Gibco) supplemented with 10% heat-inactivated fetal bovine serum (FBS) (catalog no. LSFB-0500; Equitech-Bio), 1.5 g/liter sodium bicarbonate (Sigma-Aldrich, St. Louis, MO), and 50 U/mL penicillin plus 50 mg/mL streptomycin (PenStrep, catalog no. 15140–122; Gibco) and incubated at 37 °C under a humidified 5% CO_2_ atmosphere.

Cells were passaged upon reaching 70–80% confluence (4 days after dissection for ED10.5) by aspirating old media, washing twice with 1X Phosphate-Buffered Saline (PBS) and applying 2.5% Trypsin/EDTA solution. Cells were incubated with trypsin/EDTA solution at 37 °C until they began to detach from the bottom of the culture dish. Warmed DMEM was added to neutralize trypsin activity and cells were collected and centrifuged for 5 min at 1000 x g. Following centrifugation, the supernatant was aspirated and the cell pellet was resuspended in warmed DMEM. The ED10.5 whole embryo cultures represented mouse embryonic fibroblast, typically 95% of cells in culture tested positive for fibroblast surface protein (data not shown). Since these were primary cell cultures, cells were subseeded only once, to ensure comparable sample sizes.

Twenty-four hours before treatment, cells were counted and plated at 100,000 cells/mL for treatment in pre-culture medium (5% FBS, 1%PS) to synchronize the cells. To assess cell response, cell cultures were exposed to different cell death inducers as follows: Ethanol (EtOH): 200 Proof Ethanol (Catalog# 111HPLC20S, Pharmco-AAPER, Brookfield, CT) was diluted in pre-culture medium to the desired final concentrations. The LD_50_ for ethanol was identified as 50–400 mM for all mixed cell cultures as read at 24 h.

5-Aza-2′-deoxycytidine (5-Aza-dC): (Catalog #A3656, Sigma-Aldrich, Saint Louis, MO) was dissolved in DMSO to a stock concentration of 20 mM and further diluted in pre-culture medium to 20 mM. Medium was replenished every 3 days with 5-Aza-dC supplement. ED10.5 cells, 5 × 105/ml, were incubated for 5 consecutive passages at 3–4 d each in DMEM supplemented with 10% FBS and 1% penicillin/streptomycin plus 50 μM 5-Aza-2′-dC dissolved in DMSO. Population doubling was calculated as: tD = (t − t0)log2/(log N − log N0), where tD is doubling time, t and t0 are the times at which the cells were counted, and N and N0 are the cell numbers at times t and t0, Medium was replenished every 3 d. At 24 h after the fifth passage, cells were suspended and assessed for DNA methylation. Cells were maintained in this culture medium for 5 population doublings.

5-Aza-dC + EtOH co-treatment: Cells were maintained as described above on 5-Aza-dC for 5 population doublings, then exposed to 400 mM EtOH for 24 h.

Disulfiram (DSF): Tetraethylthiuram disulfide (Catalog #T1132, Sigma-Aldrich, St. Louis, MO) was dissolved in DMSO to a concentration of 0.1 mM, and then further diluted to its final working concentration of 0.5 mM in pre-culture medium. This treatment occurred 1 h prior to any secondary treatment.

N-Acetyl Cysteine (NAC): (Catalog #ALX-105-005-G005, Enzo Life Sciences, Farmingdale, NY) was dissolved in ethanol to a concentration of 20 mM and to a final working concentration of 100 mM in pre-culture medium for 1 h before supplementing with an inducer or, treatment occurred 1 h prior to supplementing cells with secondary inducer 400 mM EtOH or 50 mM pyocyanin for a further 2, 4, 12 or 24 h.

Pyocyanin: (Catalog #ENZ-53001-C001, Enzo Life Sciences, Farmingdale, NY) was dissolved in DMSO to a stock concentration of 50 mM and to a final concentration of 50 mM in pre-culture medium.

#### Trypan blue exclusion assay

Dead and dying cells experience a loss of plasma membrane integrity, allowing vital dyes such as trypan blue to enter the cell [25]. After treatment, cells were collected at the desired time and centrifuged at 1000 x g for 5 min. The medium was aspirated and the cell pellet was resuspended in warmed 1x PBS. A 100 mL sample of this cell suspension was mixed with an equal volume of 0.4% trypan blue solution (Catalog# T6146-25G, Sigma-Aldrich, St. Louis, MO) and incubated at room temperature for 3 min. Cells were then counted on a hemocytometer under a light microscope. Each assay was run in triplicate. Data were typically expressed as percent cell death.

Since our results with trypan blue were completely consistent with other assays, such as Live/Dead® and Hoechst 33258 (Hoechst, Frankfurt, Germany) staining (data not shown), we relied primarily on this simple and reliable assay. For each experiment, this number for the experimental sample was normalized by subtracting the basal level of cell death observed in the control (9–20%) for each treatment. Statistical significance of the results was calculated by standard *t* test; values of *P* < 0.05 were considered significant.

#### MTT assay

MTT (3- (4,5-dimethylthiazol-2-yl)-2,5-diphenyltetrazolium bromide) (Catalog# M2128, Sigma-Aldrich, Saint Louis, MO) is a soluble, bright yellow compound that is reduced to insoluble purple formazan in metabolically active. MTT can also be used to measure changes in mitochondrial activity. To measure the viability of a cell population, 1.5 × 10^6^ cells were seeded into 35 mm tissue culture dishes and allowed to attach overnight at 37 °C in minimum culture medium (MCM). Media centrifuged at 1000 x g for 5 min to collect and pellet any dead, floating cells. These cells were resuspended in a small amount of media and put back into the cell culture plate. One mL of a 1:10 dilution in Maintenance Culture Medium MCM of 5 mg/mL MTT stock was then applied to each plate, which was then incubated at 37 °C for 2 h. One mL of acidic isopropanol (0.04 M HCl in absolute isopropanol) was then added to each plate and mixed thoroughly to solubilize the newly formed formazan. All cells and supernatant were then collected from each plate and centrifuged at 14,000 rpm for 5 min to remove cell debris. The supernatant from each sample was then transferred into a plastic spectrophotometer cuvette and the absorbance of the converted dye was measured at 540 nm. A decrease in absorbance indicates a decrease in mitochondrial activity and/or death [26].

WST-1 (Water Soluble Tetrazolium salts) (Catalog# 630118, Clontech, Mountain View, CA) is cell-impermeable dye and is used to measure cellular metabolic activity via NAD(P)H-dependent cellular oxidoreductase enzymes and may, under defined conditions, reflect the number of viable cells present. Treated cells were seeded into 96-well tissue culture dishes (1.5 × 10^6^ cells/well) and allowed to attach overnight at 37 °C in MCM. Cells were then treated before incubation at 37 °C in the appropriate media until the desired time. WST-1 reagent was then added to the medium in dish during the final treatment hour. The dish was then read on a microplate reader at a wavelength of 450 nm with a background subtraction at 690 nm. A decrease in absorbance indicates a decrease in mitochondrial activity and/or death [27].

#### Analysis of gene expression

Quantitative Real Time Polymerase Chain Reaction (PCR) - Small PCR products (100–200 base-pairs) were amplified in quadruple on a Roche LightCycler 2.0 real-time PCR machine, using universal PCR conditions (65C to 59C touch-down, followed by 40 cycles [15“ at 95C, 10” at 59C and 10“ at 72C]). 500 μg of cDNA was amplified in 20 μL reactions [0.3X Sybr-green, 3 mM MgCl_2_, 200 μM dNTPs, 200 μM primers, 0.5 unit Platinum Taq DNA polymerase (Roche)]. Primer-dimers were assessed by amplifying primers without cDNA. Primers were retained if they produced no primer-dimers or only non-specific signal after 40 cycles. Results were calculated as relative intensity compared to female expression. The last cycle was retained as baseline for comparison with “absent” genes. Data were plotted as “CT” (Cycle Threshold) values, in which the CT value represents the cycle at which fluorescence is first detected. By this representation, a lower cycle number indicates a higher initial concentration of mRNA, and each decrease of one cycle indicates a doubling of initial concentration [1, 2]. The primer sequences were:

**Table:** 

Gene		Primer Sequence	Product Size
Cyp2e1	FW	ACGTAAACGGCCACAAGTTC	198
	REV	AAGTCGTGCTGCTTCATGTG	
ADH I	FW	TACACCCAGTCACAATAGGAGAGTG	321
	REV	CCATGCATTCATTGTCACACTTGTGG	
ADH II	FW	TCTGCATGAAGGTCGAAGTG	256
	REV	TTCCCAAAAAGAGCACATCC	
ADH IV	FW	AGAAACTGGCTTCGGCACTA	278
	REV	CAATCCCGAAGTCCTCAAAA	
ADH VII	FW	TCTGCATGAAGGTCGAAGTG	322
	REV	AGAAACTGGCTTCGGCACTA	

### Detection of total ROS

Enzo Life Sciences’ Total ROS Detection Kit (Catalog # ENZ-51010, Enzo Life Sciences, Farmingdale, NY) is designed to directly monitor real time production of reactive oxygen and/or nitrogen species (ROS) in live cells using a microplate reader. The kit includes Oxidative Stress Detection Reagent (Green) as the major component. This non-fluorescent, cell-permeable total ROS detection dye reacts directly with a wide range of reactive species, such as hydrogen peroxide, peroxynitrite and hydroxyl radicals, yielding a green fluorescent product indicative of cellular production of different ROS.

Upon staining, the fluorescent product generated can be visualized using a standard green filter (490_Ex_/525_Em_ nm).

The procedure followed vendor’s specifications. In short, 1 h prior to cell treatment, the ROS dye is added to the culture dish; cell treatment is superimposed, and then readings are taken at specified times [28].

### Total glutathione assay

Total Glutathione (GSSG+GSH) levels were measured using the Sigma-Aldrich Glutathione Assay Kit, using manufacturer specifications. Cell pellets were normalized to total cell number including live and dead cells. Pellets were deproteinized with 3 volumes of 5%-Sulfosalicylic Acid solution (SSA), then centrifuged at 600 x g to remove precipitated proteins. Samples were then freeze thawed twice and left at 4 °C for 5 min. Samples were centrifuged at 10,000 x g for 10 min. Supernatant volumes were measured and retained as the original sample volume. Samples were then serially diluted in 5% 5-Sulfosalicylic Acid SSA; sample in SSA represented 10 mL of the working mixture, the rest presented the working mixture 150 mL (Glutathione Reductase (6 units/mL), DTNB (1.5 mg/mL), in Assay buffer (100 mM potassium phosphate buffer, pH 7.0, with 1 nM EDTA)). Reaction started upon adding 50 mL of NADPH (0.16 mg/mL), with a 5 min, room temperature incubation. Readings were performed in a plate reader at 412 nm with readings every minute for 5 min. Absorbance of a blank sample was subtracted as background.

Data was presented as nmoles of GSH per mL of sample [29].

### Statistical analysis

Statistical analysis was by Student T-TEST using Excel. *P* values greater than 0.05 represent no statistical difference between compared samples.

## Results

### Cells respond in a sex dependent manner to EtOH induced stress

Male and female Embryonic Day (ED) 10.5 and 17.5 mouse embryonic fibroblasts (MEF) respond in a sex dependent manner to several toxins such as ethanol (EtOH) and pathogens, collectively known as cell death inducers [1, 30]. Our laboratory has evaluated cell sensitivity and sex differences in many cell subpopulations. The ED10.5 MEF’s are most useful, as these cells have not been influenced by sex hormones produced by the embryos. We have examined cells from other developmental stages, as well as specific tissues including kidney, liver, lung, and neurons have been evaluated and we find similar outcomes. However, ED10.5 cells can undergo multiple passages, which are necessary to evaluate inhibition of DNA methylation. To evaluate the importance of innate sex differences before the appearance of embryonic sex hormones, we focused on cells from male and female ED10.5 whole embryos as described in Material and Methods. Cell viability was measured using the trypan blue exclusion assay to evaluate membrane integrity [25]. Approximately 10% of these cells die under normal culture conditions, independent of cell sex, allowing comparison of their responses to EtOH.

We exposed cultured ED10.5 whole-embryo cells to 400 μM ethanol over a 24 h period. Female cells are more sensitive to EtOH, resulting in 49% death compared to males at 29% death (Fig. 1b).

To validate these differences, we used the WST-1 (water soluble tetrazolium) assay, which measures conversion of tetrazolium to formazan in functioning mitochondria [27]. Cells exposed to EtOH were incubated with the WST-1 mixture for the last hour of the treatment. The samples were then compared for cell viability. At 24 h of EtOH exposure, mitochondrial activity was significantly reduced, though only approximately 10% in males compared to 65% in females, suggesting healthier or more active mitochondria in the male cells (Fig. 1c). We used the WST-1 assay simply to corroborate our results using trypan blue. Further explorations should include an evaluation of the importance of mitochondrial oxidative phosphorylation.

We further confirmed our results using MTT, which also measures formazan reduction [26] Using the MTT assay, we found that in comparison to the sex indifferent controls, ethanol lowered formazan reduction in both sexes, but more in cells from females, suggesting that female cells are more sensitive to EtOH when compared to the male counterparts (Fig. 1d).

The reduction in formazan conversion seen in these experiments is consistent with the cell death results, validating exploration of the pathways of alcohol metabolism.

### Inhibition of aldehyde dehydrogenase abolishes sex dimorphic sensitivity to ethanol by increasing male sensitivity to EtOH

We inhibited the canonical alcohol metabolic pathway by using Disulfiram (DSF), a known inhibitor of aldehyde dehydrogenase, indirectly blocking ADH activity as well, by generating buildup of acetaldehyde exposing cells to 0.5 μM disulfiram (DSF, as suggested in [31]); starting 1 h prior to and during the exposure to 400 μM EtOH for 4, 12 and 24 h. DSF alone is not toxic compared to controls (Fig. 1e). By 24 h of EtOH exposure, significantly more male and female cells die when compared to control, with more female cells dying as before. However, by 24 h, the sex dimorphic sensitivity to EtOH is abolished by DSF (Fig. 1e). This change results from an increase in the amount of cell death in the males from 30 to 50%. We therefore explored the regulation of other genes in the alcohol metabolism pathway.

### Expression and regulation of alcohol dehydrogenases class I-V are sex indifferent

**Fig. 2:**
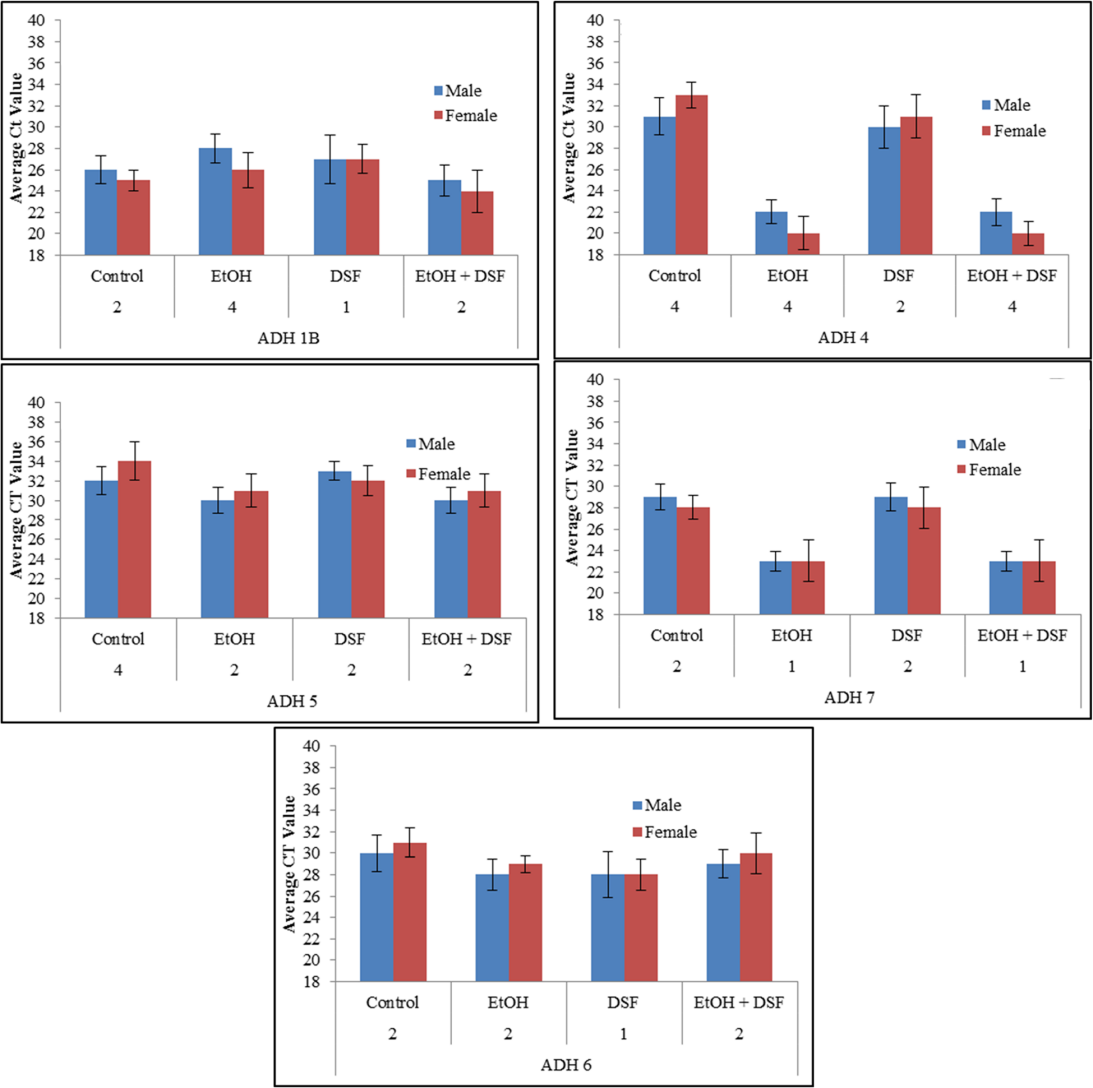
Expression Profiles for Alcohol Dehydrogenase Isoforms are not sex specific. Expression was measured by qRT-PCR using specific primers to evaluate their baseline expression. ED10.5 mixed cells. Samples were normalized at the RNA and cDNA levels for equal cDNA loading. Experiments were done in triplicate. Average CT values are shown. Ordinate is average CT of fluorescence detection for real-time PCR. ED10.5 cells exposed to 400 μM EtOH, DSF, or both for 24 h or. ADH 1B, 5, and 6 are neither responsive to EtOH nor sex dimorphic; ADH 4 and 7 are responsive to EtOH but not sex dimorphic

ED 10.5 whole embryo culture RNA was extracted and prepared for qRT-PCR for members of the ADH family as described. For alcohol dehydrogenases class I-V, we find no significant sex differences in gene expression (Fig. 2a-e). Class I ADH 1B, Class III ADH 5 and Class V ADH 6 are also unresponsive to EtOH or DSF (Fig. 2a, c and e). Class II ADH 4 and Class IV ADH 7 are responsive to EtOH exposure, with increased levels of ADH 4 and 7; this increase in expression is not reduced in the presence of DSF, and there are no sex-based differences (Fig. 2b, d), suggesting that the differences in cell sensitivity are not established by differences in alcohol dehydrogenase.

### Expressions of aldehyde dehydrogenases class I-II are sex indifferent

**Fig. 3:**
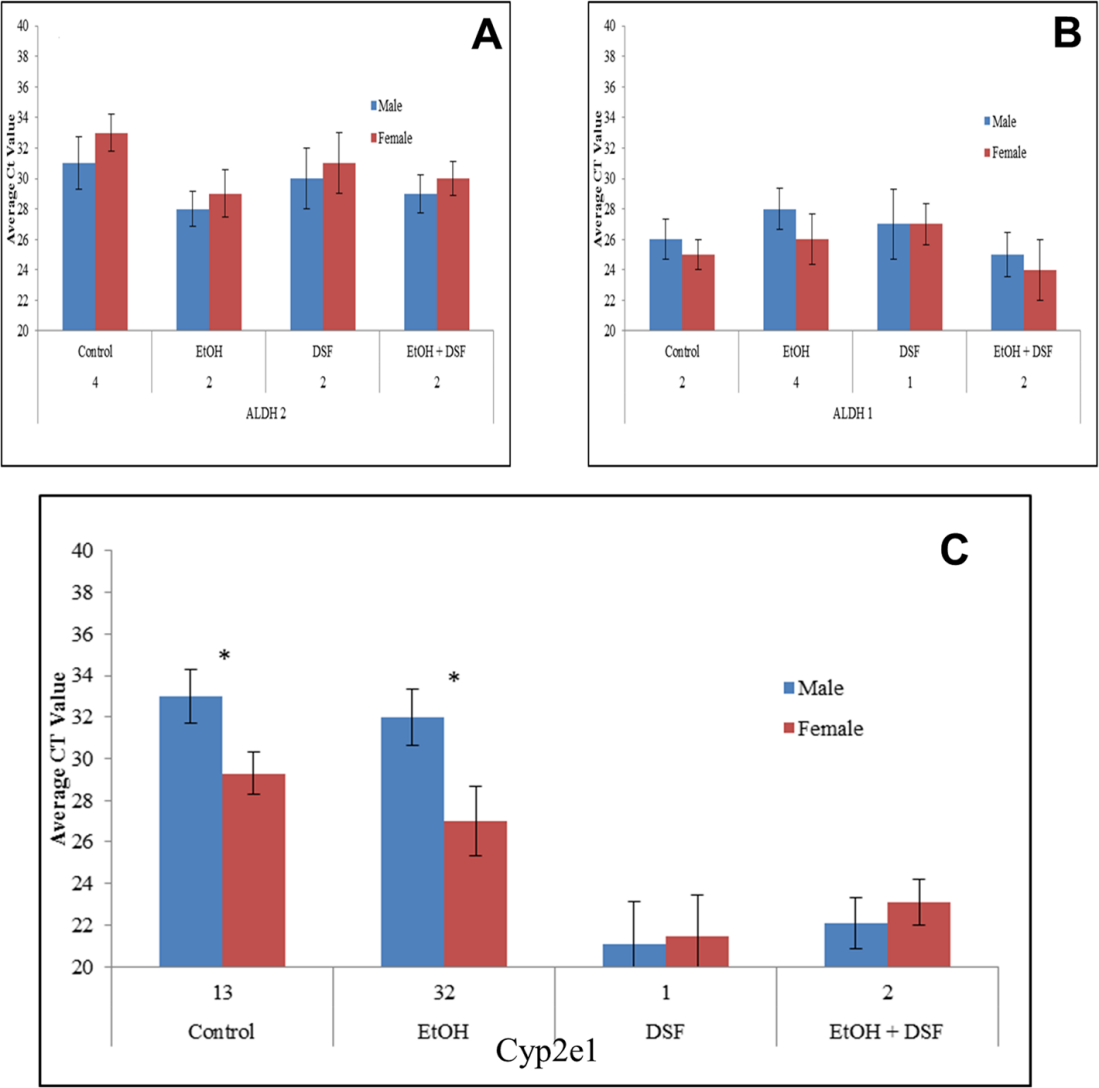
Aldehyde dehydrogenase isoforms are neither responsive to EtOH nor sex dimorphic, while Cyp2e1 is sex dimorphic and responsive. Expression in ED 10.5 cells was measured by qRT-PCR using specific primers to evaluate baseline expression. Samples were normalized at the RNA and cDNA levels for equal cDNA loading. Experiments were done in triplicate. Average CT values are shown. Ordinate is average CT of fluorescence detection for real-time PCR. ED10.5 cells exposed to 400 μM EtOH, DSF, or both for 24 h. ALDH 1 and 2 do not differ by sex or respond to EtOH, while female cells contain more Cyp2e1 message. Both male cells and female cells respond to EtOH, with the female cells responding more, increasing the differential. DSF, which inhibits aldehyde dehydrogenase, stimulates production of Cyp2e1 message in both sexes, eliminating the difference between the sexes. Asterisks (*) represent *P* < 0.05, standard deviation shown

Downstream, aldehyde dehydrogenases (ALDH) convert acetaldehyde to acetate and subsequently to acetyl-CoA (Fig. 1a). ED 10.5 whole embryo culture RNA was extracted and prepared for qRT-PCR. In the case of ALDH 1 and ALDH 2, No differences in transcription are seen for either ALDH 1 or 2: and these isoforms are not induced by EtOH, nor inhibited by (Fig. 3a-b).

We previously established that three Cyp450 family members are expressed in a sex specific manner and suggested that these could be important in detoxification [1, 2]. Because the canonical alcohol metabolic pathway did not present differences, we examined the expression and role of Cyp2e1 in the presence of ethanol.

### Gene expression of non-canonical ethanol metabolizing enzymes is sex dimorphic

At toxic alcohol levels, the non-canonical alcohol metabolic pathway is triggered, where Cyp2e1 is the active enzyme oxidizing ethanol (Fig. 1a). For cells from ED 10.5 females, Cyp2e1 transcription is 13-fold higher than for male cells, and ethanol increased this difference to 32 fold, by selectively increasing the expression in females over males. Inhibiting Aldehyde Dehydrogenase by DSF alone resulted in an up-regulation of Cyp2e1 for both male and female cells, eliminating differences in expression of Cyp2e1. Exposing cells to both DSF and EtOH resulted in an increase of Cyp2e1 but no differences between the sexes, indicating that Cyp2e1 was responsive to both EtOH and DSF (Fig. 3c).

Since Cyp2e1 is known to generate radicals, we asked whether radical generation was enough to elicit sex differences in cell survival.

### Pre-treatment with ROS scavenger, N-acetyl cysteine, reduces EtOH induced cell death

**Fig. 4:**
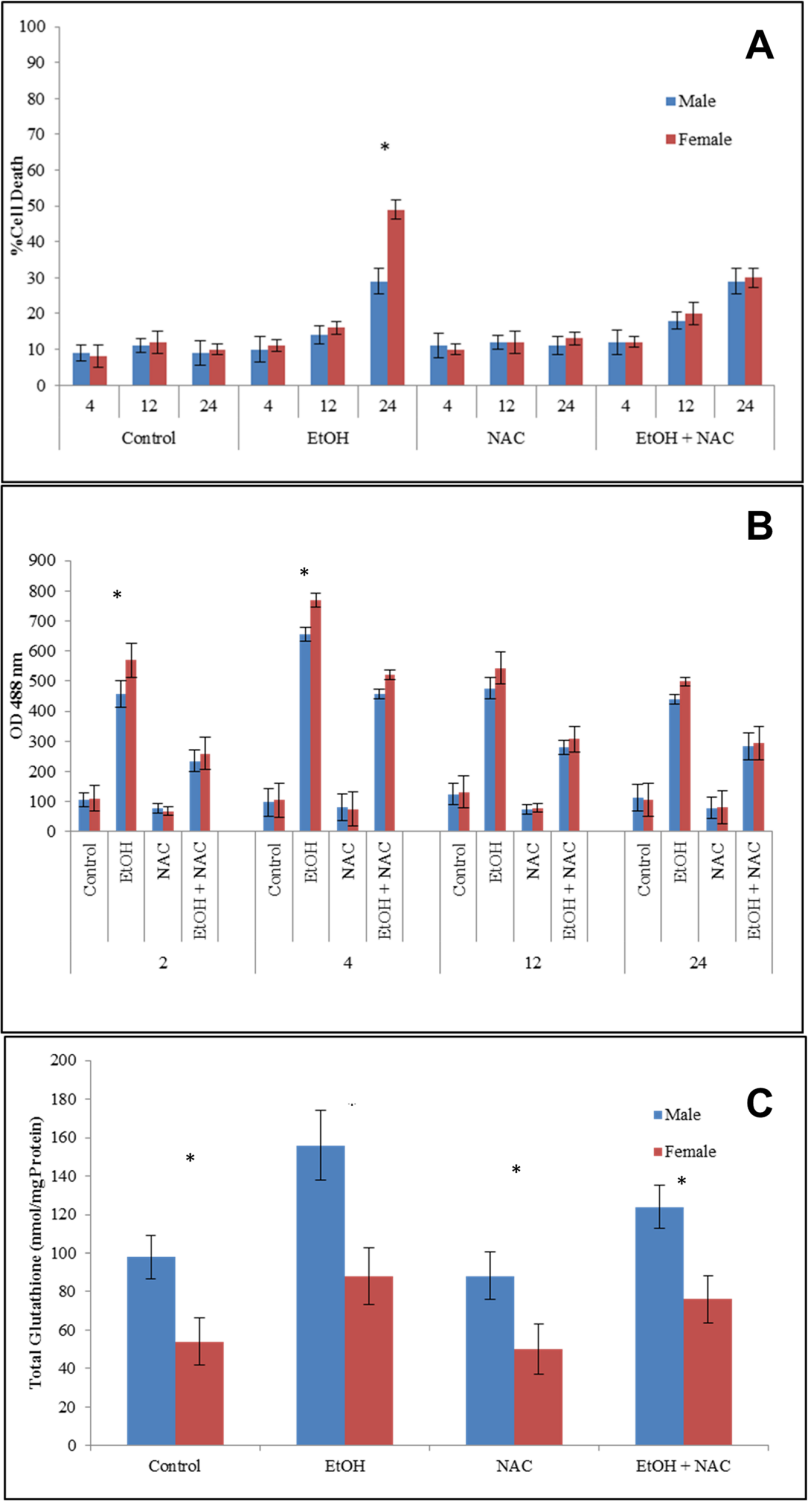
EtOH induces generation of ROS, which can be partially blocked by N-acetyl cysteine. Male cells, with higher glutathione concentrations, tolerate ROS more than female cells. **a** Cell death measured by trypan blue exclusion. Cells were exposed to 400 μM ethanol, N-Acetyl Cysteine or a combination. Female cells are more sensitive to ethanol, but not if the cells are co-treated with NAC. Asterisk (*) represents *p* < 0.05, standard deviations shown. **b** Total ROS generation was detected for all conditions. Data represent 6 replicates. ED10.5 male (blue bars) and female (red bars) were treated with EtOH for 24 h or NAC for 24 h individually, or with a 1 h pretreatment of NAC followed by co treatment with EtOH and NAC for another 24 h period. Average OD values, representing oxidized dye, are shown. ROS generation is sex independent in normal cell culture conditions, strongly induced by ethanol with females generating more ROS, and partially rescued, with loss of sex difference, by pre-treatment with NAC. Asterisks (*) represent *p* < 0.05, standard deviations are shown. **c** Total GSH concentrations were assessed. ED10.5 cells were treated with EtOH 400 μM for 24 h, or pre-treated with NAC for 1 h prior to EtOH treatment, followed by co-treatment with EtOH, or NAC alone for 24 h. GSH is higher in males under all conditions with substantial increases in EtOH-treated cells. Data represent percentage total GSH in cultures. Asterisks (*) represent *p* < 0.05, standard deviations shown

If females have more Cyp2e1, and are generally more sensitive to EtOH, differential generation of ROS can account for the difference in cell survival. We initially supplemented ED10.5 cells with a ROS scavenger, N-Acetyl Cysteine (NAC) and examined cell death. ED10.5 cells were exposed to ethanol and NAC alone or in combination. NAC exposure does not affect the basal levels of death caused by normal cell culture conditions. However, when cells are pretreated with NAC and then exposed to 400 μM EtOH for 24 h more female cells survived, eliminating the difference between the sexes (Fig. 4a), suggesting that ROS and sensitivity correlate, NAC however does not affect expression of Cyp2e1, ADH or ALDH (**Data not shown**).

The failure of NAC to eliminate cell death suggests that EtOH kills by other means as well or that the half-life of NAC is less than that for EtOH. Importantly, modulating ROS scavenging alone eliminates sex differences, but without completely eliminating the toxicity of EtOH. We therefore asked whether this toxicity is mediated through ROS. We cannot eliminate the possibility that NAC acts by supplying thiol, thus modifying the configuration, cross-linking, or interaction of other proteins. However, the data are consistent with its function as an antioxidant.

### ROS levels are sex dimorphic only after exposure to EtOH

The Total ROS/Superoxide Detection Kit (Enzo Technologies) reacts directly with a wide range of reactive species (i.e. hydrogen peroxide, peroxynitrite, and hydroxyl radicals) yielding a green fluorescent product, visible at (490_Ex_/525_Em_). Our results indicate that EtOH induces the generation of ROS.

Male and female cells have similar basal ROS, and in the presence of pyocyanin (positive control), there is a substantial increase of ROS signal in both sexes, validating the technique. Pre-treatment with NAC 1 h prior to pyocyanin treatment resulted in a substantial reduction but not depletion of ROS signal. NAC exposure alone resulted in a significant reduction of ROS in control cells, suggesting that basal conditions still result in ROS generation (**Data not shown**).

To evaluate whether EtOH alone would induce sexually dimorphic generation of ROS, we seeded ED10.5 cells into 96-well microplates pretreated some with NAC 1 h before adding EtOH and incubating further up to 24 h.

ROS generation is highest at 4 h post treatment. Female cells produce significantly more ROS than their male counterparts; and pretreatment with NAC reduced the ROS generated, significantly reducing but not totally eliminating the sex differences. Since ROS were not persistent, we evaluated ROS for EtOH-exposed cells at 12 and 24 h. At these times, ROS are reduced but nevertheless high. ROS are very unstable; we have no further explanation for the transience (Fig. 4b). Nevertheless, the substantial difference at 4 h correlates directly to the dimorphic lethality, measured at 24 h (Fig. 4a), suggesting that ROS ultimately kills the cells.

### Endogenous ROS scavenging mechanisms are higher in males

Glutathione (GSH) is an endogenous scavenger of free radicals, quenching them by redox reactions. GSH is responsive to intracellular free radicals. GSH concentrations before and after generation of ROS can suggest where differences between the sexes exist.

Male and female ED10.5 cells were used to determine total GSH by assay for NADPH conversion, an indirect method of measuring total GSH. Under normal culture conditions, male cells had more total GSH, and EtOH increased GSH in both, while still maintaining the sex differences (Fig. 4c), with males having more total GSH. In all assessed conditions male cells have higher GSH (Fig. 4c), suggesting that male cells are better able to cope with EtOH induced stress perhaps by differential inactivation of ROS.

These data indicate that ethanol toxicity derives from generated ROS, with males being protected more by having higher GSH and less Cyp2e1. We thus turned to the possibility that methylation differences determine the amount of Cyp2e1 in both sexes.

### Sex specific DNA methylation results in differential Cyp2e1 transcription and EtOH induced death

**Fig. 5:**
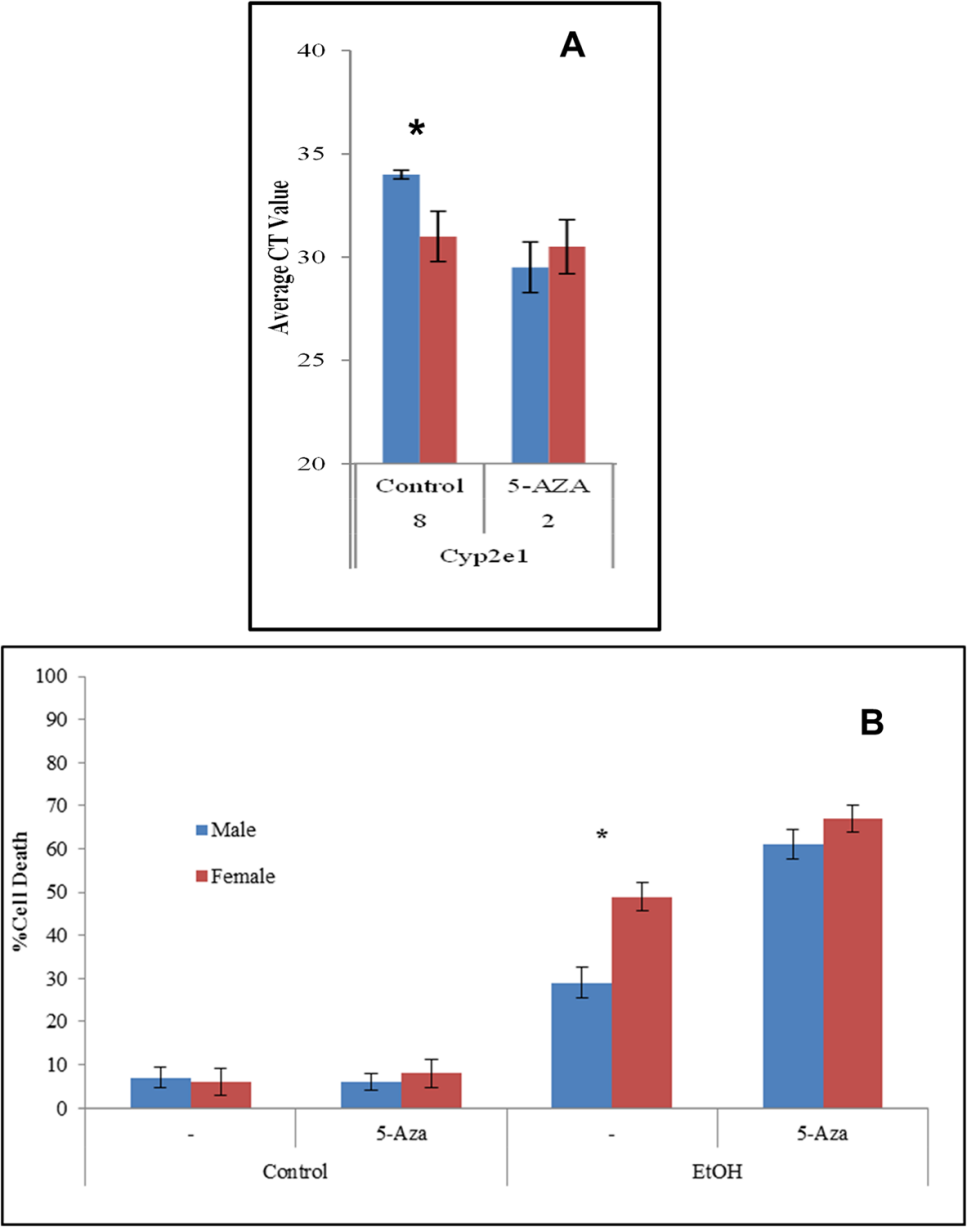
5-Aza-dC reduces sex differences in expression of Cype2e1 and makes cell more sensitive to EtOH. **a** The inhibitor of methylation increases expression of Cyp2e1 while reducing the difference in expression of Cyp2e1 from M/F = 8 to M/F = 2. Samples were normalized at the RNA and cDNA levels for equal cDNA loading. Asterisks (*) represent *p* < 0.05; standard deviations shown. **b** Cell death measured by trypan blue exclusion. Blue bars, male; red bars, females. ED 10.5 cells were exposed to 400 μM ethanol, 5-Aza-dC or a combination. Female cells are more sensitive to EtOH; 5-Aza-dC is toxic but eliminates the sex difference. Asterisk (*) represents *p* < 0.05, standard deviation shown

Blocking de novo DNA methylation effectively reduced differences in expression of several genes that showed sex dimorphisms, including Cyp2e1 [2]. The DNA methyltransferase inhibitor 5-aza-2-deoxycytidine (5-Aza-dC) reduces the difference in the expression of Cyp2e1 (Fig. 5a); we therefore tested its effect on cell viability. We used ED10.5 cells exposed to 5-Aza-dC for 5 population doublings as described in Materials and Methods, followed by exposure to 400 μM EtOH for 24 h, and assessed cell viability.

Pre-treatment with 5-Aza-dC eliminates the difference in sensitivity to EtOH, with a greater increase in death among males. Reduced de novo DNA methylation effectively increased cell sensitivity, measured by cell death increasing from 30 to 59% in males and from 49 to 65% in females, in effect reducing previously reported sex differences in response to EtOH by increasing death in both sexes (Fig. 5b).

## Discussion

Differences between male and female cells in sensitivity to EtOH persist whether or not endogenous sex hormones are present, as demonstrated in cell cultures from embryos prior to onset of sex hormone production [1]. This difference correlates with a differential methylation of the regulatory CpG islands for cytochrome p450 genes [2].

Here we investigate the biochemical mechanism of the dimorphism, using the metabolism of ethanol as a controllable stress. Of the two primary means of metabolizing ethanol (Fig. 1A), the non-canonical pathway (red) is a two-step process regulated by cyp4502e1, a member of the cytochrome P450 member family that metabolizes alcohol into acetaldehyde and then acetate. This non-canonical pathway occurs in microsomes, and therefore is triggered only at high (toxic) concentrations of EtOH; it generates free radicals, and therefore is not the most efficient mechanism.

Female cells are more sensitive to EtOH exposure (Fig. 4a), and this differential sensitivity correlates with the expression of Cyp2e1 (3C) as well as generation of ROS (Fig. 4b). The expressions of ADH and ALDH (Figs. 2 and 3a-b) do not correlate with the differential sensitivity observed (1B-D). An ADH inhibitor, DSF, renders cells, especially male cells, more sensitive to EtOH, suggesting that the non-canonical pathway compensates, resulting in more ROS and therefore more cell death (Fig. 1E). Terasaki et al. [32], reviewing reports of fetal alcohol syndrome, found little suggestion of sex differences, particularly anatomical, but emphasized that these reports generally did not examine more subtle differences in function of various regions of the brain. Converse et al. [33], on the other hand, found that giving moderate amounts of alcohol to pregnant Rhesus monkeys led to measurable sex-based differences in dopamine D1 receptors in the mature progeny.

Using NAC as a ROS scavenger allowed us to test whether it was possible ROS generated through cell stress to EtOH [34]. Female cells first exposed to NAC had reduced sensitivity to ethanol (Fig. 4a); EtOH alone elicited an increase in ROS in both males and females, but higher in females. NAC was insufficient to eliminate the ROS generated by exposure of cells to EtOH, but did sufficiently reduce ROS to levels where we no longer observed differences between males and females (Fig. 4b).

Cyp2e1 affects metabolism of toxic-level alcohol, producing ROS as a byproduct. We therefore blocked de novo methylation of DNA, which we had previously documented to alter Cyp2e1 expression [2]. Here we find that blocking DNA methylation eliminated sex differences in cellular sensitivity to EtOH, while having a greater impact in male cells. Sex differences may also be generated by miRNAs coded by the X chromosome that can affect the activity of genes located on other chromosomes [35]. We also examined other estrogen response elements [1]. Since methylation appears to be an important element here, we favor the argument of the importance of methylation, but other interactions have not been excluded.

**Fig. 6:**
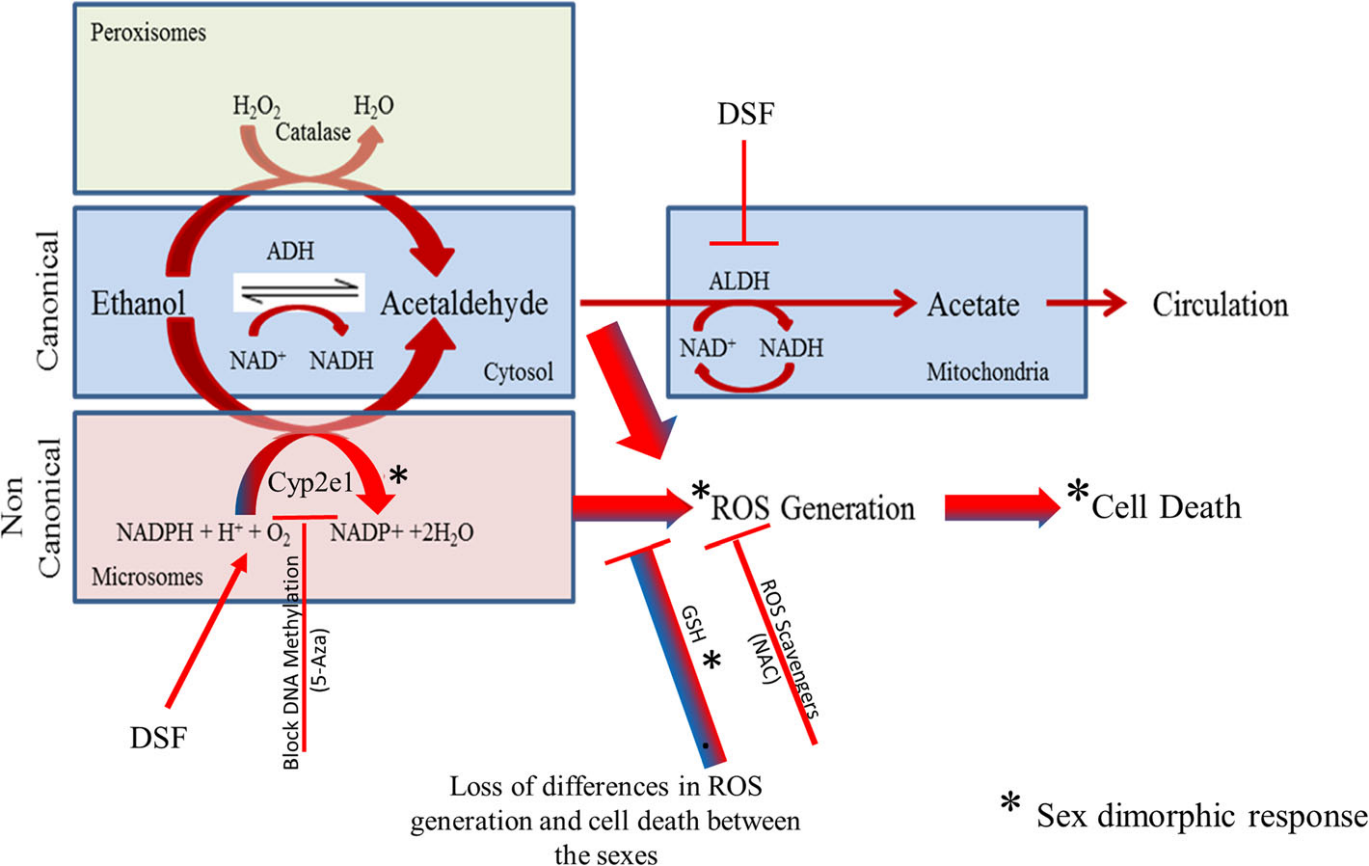
Role of Cyp2e1 in sex differences of the ethanol degradation pathway. Female cells are more sensitive to cell death by ethanol. We propose that female cells express more Cyp2e1, owing to lesser methylation of CpG islands in the gene, while male cells maintain higher GSH and GSH responsiveness. These two differences allow greater generation of ROS, and hence higher susceptibility to EtOH. Relative importance of the differences are indicated by predominance of red (female) in three branches of the pathway and the predominance of blue (male) in the role of GSH

These findings suggest that at basal levels, male and female ED10.5 cells have different levels of Cyp2e1, as well as glutathione. Female cells are significantly more susceptible to toxic concentrations of EtOH, resulting in higher ROS production and ultimately -more cell death. Inhibition of the canonical alcohol metabolizing pathway eliminates the sex differences, as a result of increased ROS production and increase in cell death in males. Scavenging ROS reduces cell sensitivity to EtOH. The results also suggest that much of the EtOH induced cell death derives from production of ROS, which can be modulated by scavenging ROS with NAC, or by modulating of Cyp2e1, the expression of which is impacted by DNA methylation (Fig. 6). It will be important to explore these options by exposing pregnant rodents to ethanol, or by more carefully collecting clinical data.

## Conclusions

We conclude that alcohol induced death resulted from generation of reactive oxygen species (ROS), potentially from Cyp2e1 activity, and that differences in Cyp2e1 resulted in the sex differences observed in cell viability. We reviewed sex differences in the alcohol metabolism pathway when methylation of DNA was blocked. Alcohol dehydrogenase and aldehyde dehydrogenase are not sex dimorphic and while their expression can be modulated by alcohol, this modulation is not sex specific. Only Cyp2e1, performing a redundant role to that of alcohol dehydrogenase responded. Loss of differences in Cyp2e1, combined with exposure to alcohol, resulted in increased death in males, effectively eliminating differences between the sexes in cell viability.

These results are best explained as generation by Cyp2e1 of radicals at toxic alcohol concentrations. Under basal and ethanol induced conditions female cells express more Cyp2e1, potentially resulting in higher generation of ROS. ROS scavenger N-Acetyl-Cysteine (NAC) reduces ethanol induced death and the differences between the sexes. Finally, DSF (Disulfiram), which blocks ALDH, increases death in both sexes as well, possibly because the increased acetaldehyde can be metabolized by Cyp2e1. Death induced by ethanol is mediated by ROS generation, which can be partially prevented by NAC, the latter of which does not distinguish by sex.

We can characterize sex differences at the cellular level; differences observed in response to ethanol as a stimulant are likely due to differences in cytochrome p450 member 2e1; and the ROS generation mediated by Cyp2e1 is the potential proximate cause of the toxicity. Blocking DNA methylation alone reduces the observed differences in cell viability, gene expression and ROS generation induced by alcohol. This is the first instance where sex differences in alcohol metabolism are described at a molecular level and suggesting that DNA methylation is one of the initial mediators of this response.

## Data Availability

All data and results not presented here are available upon request to corresponding author.

## Abbreviations

5-Aza-dC: 5-Aza-2′-deoxycytidine
ADH: Alcohol dehydrogenase
ALDH: Aldehyde dehydrogenase
CO_2_: Carbon dioxide
CYP: Cytochrome protein
Cyp2e1: Cytochrome protein 2 variant e1
CT: Cycle threshold
dH_2_O: Distilled water
DMEM: Dulbecco’s Modified Eagle Medium
DNA: Deoxyribonucleic acid
dNTP: Nucleotides (deoxynucleotide phosphate)
DSF: Disulfiram
DTNB: 5,5′-Dithiobis-(2-Nitrobenzoic Acid)
ED: Embryonic day
EDTA: Ethylene diamine tetraacetic acid
ERK: Extracellular signal-related kinase
EtOH: Ethanol
FBS: Fetal bovine serum
GSH: Glutathione
GSSG: Oxidized glutathione
hPXR: Pregane X receptor-Humanized mice
MCM: Minimal culture medium
MEF: Mouse embryonic fibroblasts
MEOS: Microsomal ethanol oxidizing system
mRNA: Messenger RNA
MTT: 4,5-dimethylthiazol-2-yl)-2,5-diphenyltetrazolium bromide
NAC: N-acetyl cysteine
NAD(P)H: Nicotine adenine dinucleotide (phosphate)
PBS: Phosphate buffered saline
PCR: Polymerase chain reaction
PenStrep: Penicillin plus streptomycin
qRT-PCR: Quantitative real time polymerase chain reaction
QTL: Quantitative trait loci
ROS: Reactive oxygen species
SSA: Sulfosalicylic acid
Taq: Thermophilus aquaticus
WST-1: Water Soluble Tetrazolium salts

## References

[1] Penaloza C, Estevez B, Orlanski S, Sikorska M, Walker R, Smith C (2009). Sex of the cell dictates its response: differential gene expression and sensitivity to cell death inducing stress in male and female cells. FASEB J.

[2] Penaloza CG, Estevez B, Han DM, Norouzi M, Lockshin RA, Zakeri Z (2014). Sex-dependent regulation of cytochrome P450 family members Cyp1a1, Cyp2e1, and Cyp7b1 by methylation of DNA. FASEB J.

[3] Werck-Reichhart D, Feyereisen R (2000). Cytochromes P450: a success story. Genome Biol.

[4] Skaanild MT, Friis C (1999). Cytochrome P450 sex differences in minipigs and conventional pigs. Pharmacol Toxicol.

[5] Lu YF, Jin T, Xu Y, Zhang D, Wu Q, Zhang YK (2013). Sex differences in the circadian variation of cytochrome p450 genes and corresponding nuclear receptors in mouse liver. Chronobiol Int.

[6] Barnett CR, Rudd S, Flatt PR, Ioannides C (1993). Sex differences in the diabetes-induced modulation of rat hepatic cytochrome P450 proteins. Biochem Pharmacol.

[7] Suto JI, Kojima M (2018). Quantitative trait loci that determine plasma insulin levels in F2 intercross populations produced from crosses between DDD/Sgn and C57BL/6J inbred mice. J Genet.

[8] Hu Z-Y, Zhao Y-S (2010). Sex-dependent differences in cytochrome P450 3A activity as assessed by midazolam disposition in humans: a meta-analysis. Drug Metab Dispos.

[9] Millecam J, De Clerck L, Govaert E, Devreese M, Gasthuys E, Schelstraete W (2018). The ontogeny of cytochrome P450 enzyme activity and protein abundance in conventional pigs in support of preclinical pediatric drug research. Front Pharmacol.

[10] Naidoo P, Chetty M (2019). Progress in the consideration of possible sex differences in drug interaction studies. Curr Drug Metab.

[11] Tamargo J, Rosano G, Walther T (2017). Gender differences in the effects of cardiovascular drugs. Eur Heart J Cardiovasc Pharmacother.

[12] Crabb DW, Bosron WF, Li TK (1987). Ethanol metabolism. Pharmacol Ther.

[13] Lu Y, Cederbaum AI (2010). CYP2E1 potentiation of LPS and TNFα-induced hepatotoxicity by mechanisms involving enhanced oxidative and nitrosative stress, activation of MAP kinases, and mitochondrial dysfunction. Genes Nutr.

[14] Lu Y, Cederbaum AI (2008). CYP2E1 and oxidative liver injury by alcohol. Free Radic Biol Med.

[15] Beisswenger TB, Holmquist B, Vallee BL (1985). chi-ADH is the sole alcohol dehydrogenase isozyme of mammalian brains: implications and inferences. Proc Natl Acad Sci.

[16] Haseba T, Ohno Y (2010). A new view of alcohol metabolism and alcoholism—role of the high-km class III alcohol dehydrogenase (ADH3). Int J Environ Res Public Health.

[17] Pares A, Caballeria J, Bruguera M, Torres M, Rodes J (1986). Histological course of alcoholic hepatitis. Influence of abstinence, sex and extent of hepatic damage. J Hepatol.

[18] Spruiell K, Gyamfi AA, Yeyeodu ST, Richardson RM, Gonzalez FJ, Gyamfi MA (2015). Pregnane X receptor-humanized mice recapitulate gender differences in ethanol metabolism but not hepatotoxicity. J Pharmacol Exp Ther.

[19] Fernandez-Abascal J, Ripullone M, Valeri A, Leone C, Valoti M (2018). beta-Naphtoflavone and ethanol induce cytochrome P450 and protect towards MPP(+) toxicity in human neuroblastoma SH-SY5Y cells. Int J Mol Sci.

[20] Kunitoh S, Imaoka S, Hiroi T, Yabusaki Y, Monna T, Funae Y (1997). Acetaldehyde as well as ethanol is metabolized by human CYP2E1. J Pharmacol Exp Ther.

[21] Qi XM, Miao LL, Cai Y, Gong LK, Ren J (2013). ROS generated by CYP450, especially CYP2E1, mediate mitochondrial dysfunction induced by tetrandrine in rat hepatocytes. Acta Pharmacol Sin.

[22] Heber-Katz E, Chen P, Dvm LC, Zhang X-M, Troutman S, Blankenhorn EP (2004). Regeneration in MRL mice: further genetic loci controlling the ear hole closure trait using MRL and M.m. Castaneus mice. Wound Repair Regen.

[23] French SW, Morimoto M, Reitz RC, Koop D, Klopfenstein B, Estes K (1997). Lipid peroxidation, CYP2E1 and Arachidonic acid metabolism in alcoholic liver disease in rats. J Nutr.

[24] Gonzalez FJ, Gelboin HV (1990). Transcriptional and posttranscriptional regulation of CYP2E1, an N-nitrosodimethylamine demethylase. Princess Takamatsu Symp.

[25] Allison DC, Ridolpho P (1980). Use of a trypan blue assay to measure the deoxyribonucleic acid content and radioactive labeling of viable cells. J Histochem Cytochem.

[26] Meerloo J, Kaspers GL, Cloos J. Cell sensitivity assays: the MTT assay. In: Cree IA, editor. Cancer cell culture. Methods in molecular biology. 731. Totawa: Humana Press; 2011. p. 237–45. 10.1007/978-1-61779-080-5_20 21516412

[27] Ngamwongsatit P, Banada PP, Panbangred W, Bhunia AK (2008). WST-1-based cell cytotoxicity assay as a substitute for MTT-based assay for rapid detection of toxigenic Bacillus species using CHO cell line. J Microbiol Methods.

[28] Jambunathan N (2010). Determination and detection of reactive oxygen species (ROS), lipid peroxidation, and electrolyte leakage in plants. Methods Mol Biol.

[29] Romagnoli C, Marcucci T, Picariello L, Tonelli F, Vincenzini M, Iantomasi T (2013). Role of N-acetylcysteine and GSH redox system on total and active MMP-2 in intestinal myofibroblasts of Crohn's disease patients. Int J Color Dis.

[30] Nikezic-Ardolic M, Lin L, Milcevic M, Zakeri Z (1999). Gender differences in cellular response. Lupus.

[31] Lipsky JJ, Shen ML, Naylor S (2001). In vivo inhibition of aldehyde dehydrogenase by disulfiram. Chem Biol Interact.

[32] Terasaki LS, Gomez J, Schwarz JM (2016). An examination of sex differences in the effects of early-life opiate and alcohol exposure. Philos Trans R Soc Lond Ser B Biol Sci.

[33] Converse AK, Moore CF, Holden JE, Ahlers EO, Moirano JM, Larson JA, Resch LM, DeJesus OT, Barnhart TE, Nickles RJ, Murali D, Christian BT, Schneider ML (2014). Moderate level prenatal alcohol exposure induces sex differences in dopamine D1 receptor binding in adult rhesus monkeys. Alcohol Clin Exp Res.

[34] Ferreira Seiva FR, Amauchi JF, Ribeiro Rocha KK, Souza GA, Ebaid GX, Burneiko RM (2009). Effects of N-acetylcysteine on alcohol abstinence and alcohol-induced adverse effects in rats. Alcohol (Fayetteville, NY).

[35] Matarrese P, Tieri P, Anticoli S (2019). X-chromosome-linked miR548am-5p is a key regulator of sex disparity in the susceptibility to mitochondria-mediated apoptosis [published correction appears in Cell Death Dis. 2019 Nov 4;10(11):828]. Cell Death Dis.

